# Fibroblast Growth Factor-1 Improves Insulin Resistance *via* Repression of JNK-Mediated Inflammation

**DOI:** 10.3389/fphar.2019.01478

**Published:** 2019-12-05

**Authors:** Lei Fan, Linchao Ding, Junjie Lan, Jianlou Niu, Yiling He, Lintao Song

**Affiliations:** ^1^Jinhua Hospital of Zhejiang University and Jinhua Municipal Central Hospital, Jinhua, China; ^2^School of Pharmacy, Wenzhou Medical University, Wenzhou, China

**Keywords:** fibroblast growth factor-1, diabetes, insulin sensitivity, cytokines, JNK signalling, inflammation

## Abstract

Insulin resistance is associated with a greatly increased risk of type 2 diabetes. Administration of fibroblast growth factor-1 (FGF-1) resulted in a marked improvement in insulin sensitivity. However, the underlying molecular mechanism whereby FGF-1 represses insulin resistance remains largely unknown. Here, we sought to delineate the role of FGF-1 in insulin resistance with respect to its anti-inflammatory capability. In this study, we found that FGF-1 had positive effects on glucose intolerance, hepatic lipid accumulation, and insulin resistance, while it markedly repressed cytokine secretion (TNF-α and IL-6) in serum and reduced liver inflammation in diet-induced obesity (DIO) mice. Further, FGF-1 treatment significantly represses TNF-α-induced insulin resistance *in vitro* and *in vivo*. These results indicate that FGF-1 likely ameliorates insulin resistance *via* a mechanism that is independent of its glucose-lowering activity. Subsequent experiments demonstrated that FGF-1 ameliorated insulin resistance, and inflammation was accompanied by decreased c-Jun N-terminal kinase (JNK) signaling. In addition, it is likely that FGF-1 impedes JNK phosphorylation *via* blocking the transforming growth factor-β activated kinase 1 (TAK1) and TAK1 binding protein 1 (TAB1) interaction. These findings reveal that FGF-1 regulates insulin sensitivity and may represent an attractive therapeutic target for preventing the development of insulin resistance.

## Introduction

Insulin resistance is a complex metabolic syndrome that occurs in the vast majority of diabetes (Kahn et al., 2006). The etiology of insulin resistance has been intensely studied, and chronic inflammation is recognized as an important cause of insulin resistance (Dandona et al., 2004; Shoelson et al., 2006; Wu and Ballantyne, 2017). The pro-inflammatory cytokine TNF-α is reportedly increased in obese mice, and inhibition of this cytokine improved both glucose tolerance and insulin sensitivity (da Costa et al., 2016; Shimobayashi et al., 2018). TNF-α is also a key mediator in hepatic insulin resistance (Feinstein et al., 1993; Hotamisligil et al., 1994). Primary hepatocytes and C57BL/6J wild-type mice treated with TNF-α exhibited marked hepatic insulin resistance (Hotamisligil et al., 1994; Hotamisligil and Spiegelman, 1994; Tilg and Hotamisligil, 2006). In addition, increased production of pro-inflammatory cytokines is linked to systemic inflammation and is accompanied by insulin resistance (Arkan et al., 2005; Odegaard and Chawla, 2013). Pro-inflammatory cytokines, such as TNF-α and IL6, stimulate both the c-Jun N-terminal kinase (JNK) and IκB kinase-β (IKK-β)/nuclear factor-κB (NF-κB) pathways, resulting in upregulation of potential mediators of inflammation that can lead to insulin resistance (Hirosumi et al., 2002; Klover et al., 2003; Sabio and Davis, 2010; Wan and Lenardo, 2010). In particular, JNK activation is correlated with macrophage accumulation and loss of insulin function (Hirosumi et al., 2002; Han et al., 2013). Hence, upregulation of pro-inflammatory signaling pathway is considered a key contributor to the progression of insulin resistance.

Fibroblast growth factor-1 (FGF-1), a member of the FGF family, is involved in the regulation of diverse physiological processes such as mammalian development, angiogenesis, wound healing, adipogenesis, and neurogenesis (Banai et al., 1991; Beenken and Mohammadi, 2009; Ornitz and Itoh, 2015). Paracrine FGF-1 requires heparan sulfate glycosaminoglycans to bind, dimerize, and activate their cognate fibroblast growth factor receptors (FGFRs) (Dailey et al., 2005; Goetz and Mohammadi, 2013). Activated FGFRs phosphorylate specific tyrosine residues that mediate interaction with cytosolic adaptor proteins and the RAS-MAPK, PI3K-AKT, and PLC intracellular signaling pathways, and then regulates a plethora of functions including cell growth, proliferation, migration differentiation, and survival in different cell types (Beenken and Mohammadi, 2009). Although FGF-1 is known as a mitogenic factor (Goetz and Mohammadi, 2013), Jonker and his colleagues discovered an essential role for FGF-1 in the adaptive remodeling of adipose tissue in response to nutrient fluctuations (Jonker et al., 2012). Earlier studies have generated a great deal of interest in the insulin sensitizing effects of FGF-1 in diabetic rodents. It is reported that, with a standard chow diet, *Fgf-1^-/-^* mice did not exhibit metabolic or histological abnormalities (Miller et al., 2000). However, when fed a high-fat diet (HFD), *Fgf-1^-/-^* mice developed an exaggerated diabetic phenotype, accompanied by severe insulin resistance and marked inflammation (Jonker et al., 2012). In a follow-up study, treatment with exogenous recombinant FGF-1 resulted in insulin sensitization, evidenced by suppression of hepatic glucose production and increased insulin-dependent glucose uptake (Suh et al., 2014; Huang et al., 2017). Moreover, intracerebroventricular (ICV) injection of exogenous FGF-1 suppresses hypothalamic-pituitary-adrenal axis-mediated lipolysis and hepatic glucose production in a rat model of diabetes (Perry et al., 2015; Scarlett et al., 2016). These integrative physiological observations indicate that FGF-1 is an essential growth factor for attenuating insulin resistance and metabolic disorder. However, the underlying molecular mechanism whereby FGF-1 inhibits insulin resistance remains poorly understood.

Among the many potential pathogenic mechanisms responsible for the development of insulin resistance, inflammation is recognized as a central factor driving this process (Shoelson et al., 2006). Treatment with FGF-1 reportedly remarkably lowered levels of several serum inflammatory cytokines and impeded the inflammatory response (Suh et al., 2014; Liang et al., 2018). In addition, this corresponded to the anti-inflammatory effects of FGF-1 with respect to its ability to significantly prevent the development of nonalcoholic fatty liver disease (NAFLD) and diabetic nephropathy (DN) (Liu et al., 2016; Liang et al., 2018). These findings suggest that FGF-1 has the potential to reduce inflammation; however, it remains unclear whether FGF-1-associated improvement of insulin resistance is dependent upon its anti-inflammatory effects.

In the present study, we investigated the effects of administering recombinant FGF-1 to obesity- or TNF-α-induced insulin resistance mouse models, and our findings indicated that FGF-1 significantly improves insulin resistance and reduces inflammation. Mechanistically, we found that FGF-1 improves insulin resistance and inflammation accompanied by attenuation of the c-Jun N-terminal kinase signaling pathway. To our knowledge, these findings provide the first direct experimental evidence demonstrating the protective effects of FGF-1 are linked to inflammation in the pathogenesis of insulin resistance.

## Material and Methods

### Animal Experiments

All animal procedures were performed with an approved protocol by the Institutional Animal Care and Use Committee of Wenzhou Medical University. C57BL/6J male mice were housed in a temperature controlled environment (12 h light/dark cycle) with free access to water and food. For HFD feeding, male 6-week-old C57BL/6J mice were placed on a HFD (60% fat). After 12 weeks, diet-induced obesity (DIO) mice were then randomized based on their body weight and plasma glucose level: one group of mice was subcutaneously injected with 0.1 mg/kg wild-type FGF-1 (Novoprotein, Cat. CH53), while the other group was treated with phosphate buffered saline (PBS) and served as control mice. In addition, the mice fed normal chow diet (ND) were treated with PBS as the model control. Blood glucose values were determined using OneTouch UltraVue automatic glucometers (Johnson & Johnson). Plasma insulin (ALPCO, 80-INSMSU-E01), TNF-α (R&D, MTA00B), and IL-6 (R&D, M6000B) levels were measured according to the manufacturer’s instructions. Levels of triacylglycerol (290-63701, Wako, Osaka, Japan) were determined according to the manufacturer’s instructions. For *in vivo* AKT phosphorylation detection, mice were fasted 6 h before insulin (5 U/kg, Lilly) or saline injection. For TNF-α treatment, chronic TNF-α exposure was performed as previously described (Hotamisligil et al., 1994; Peraldi et al., 1996). In brief, 12-week-old mice were implanted with pumps with a 7-day pumping capacity and an infusion rate of 1 µl/h. Pumps were filled to capacity with 7.1 µg/ml of TNF-α diluted in the carrier (0.9% NaCl and 0.1% BSA). Mice were treated with PBS or 0.5 mg/kg FGF-1 subcutaneous injection every day throughout the experiment. For histological analysis, mouse liver tissues were harvested and fixed in 4% paraformaldehyde and embedded in paraffin. Five micron sections were used for hematoxylin and eosin (H&E) staining according to the manufacturer’s instructions. For the glucose tolerance test (GTT), mice were fasted for 16 h and then injected with glucose (2 g/kg, intraperitoneally). For the insulin tolerance test (ITT), mice were fasted for 3 h and then injected with insulin (1 U/kg, intraperitoneally).

### RNA Isolation and RT-PCR

Total RNA was extracted from livers and cultured cells using TRIzol reagent (Invitrogen, 15596018) according to the manufacturer’s instructions and quantified with the use of the Nanodrop ND-2000 system. 5 µg total RNA was used to synthesize first-strand complimentary DNA (cDNA) using reverse transcription kit (Promega, WI, USA). The cDNA was then diluted and used as the template for the detection of the different transcripts. The expression levels were analyzed by quantitative real-time polymerase chain reaction (RT-PCR) on Step One Plus RT-PCR system (Applied Biosystems, USA) using SYBR Green qPCR SuperMix-UDG kit (Invitrogen). RT-PCR was performed for 3 min at 95°C, and a total of 40 cycles of 10 s at 95°C, 20 s at 60°C, and 30 s at 72°C. The ratio of the expression levels of tested genes were analyzed according to the 2^−ΔΔCT^ method and normalized to mRNA levels of Actin. The sequences of the primers, as synthesized by Invitrogen, are shown in **Table S1**.

### Hyperinsulinemia–Euglycemic Clamp in DIO Mice

Male 6-week-old C57BL/6J mice were on 60% fat HFD for 12 weeks and were subcutaneously injected with PBS and FGF-1 (0.1 mg/kg body weight) every day for 4 weeks before clamp studies. Mice hyperinsulinemic-euglycemic clamps were performed as previously described (Suh et al., 2014). Briefly, mice with indwelling catheters were fasted for 6 h and received a euglycemic clamp with continuous infusion of insulin (8 mU/kg/min, Biosharp). Glucose (D-[3-^3^H] glucose, 0.09 mCi/min) was infused at variable rates to maintain euglycemia. The glucose flow rate was adjusted to reach a steady-state blood glucose concentration. Insulin-stimulated whole-body glucose flux was estimated using continuous infusion of Glucose (D-[3-^3^H] glucose, 0.09 mCi/min) throughout the clamp procedure. Blood was collected from the tail vein at 10 min intervals during the last 30 min. At steady state, the rate of glucose disappearance or the total glucose disposal rate is equal to the sum of the rate of endogenous or hepatic glucose production and the rate of exogenous glucose infusion. The insulin-stimulated glucose disposal rate is equal to the total glucose disposal rate minus the basal glucose turnover rate.

### Antibodies and Drugs

Antibodies to phospho-Ser473-Akt (Cat. #4060), phospho-Thr308-Akt (Cat. #4056), AKT1 (Cat. #2938), phospho-Thr183/Tyr185-JNK (Cat. #4668), TAK1 (Cat. #5206), and TAB1 (Cat. #3226) were purchased from Cell Signaling Technology. Antibodies to hemagglutinin (HA; F-7) and JNK (C-17) were purchased from Santa Cruz Biotechnology. Antibodies to Flag (Cat. F2555) and Actin (Cat. A8481), anti-Flag beads (Cat. M8823), and lipopolysaccharide (LPS, Cat. L2630) were purchased from Sigma-Aldrich. Recombinant human FGF-1 (Cat. CH53) and TNF-α (Cat. CF09-B) were obtained from Novoprotein. Triglyceride assay kit (Wako, 290-63701) was purchased from FUJIFILM Wako Pure Chemical Corporation.

### Cell Culture, Transfection, and Immunoprecipitation

HEK293T and RAW 264.7 cells were maintained in DMEM containing 10% FBS, 100 IU penicillin and 100 µg/ml streptomycin in a humidified incubator with 5% CO_2_ at 37°C. For transfection, HEK293T cells were plated in 60 mm culture dishes the day before transfection. Polyethylenimine (PEI, Polysciences, 23966) at a final concentration of 10 µM was used to transfect HEK293T cells. Transfected cells were harvested 24 after transfection. For immunoprecipitation, proteins were extracted from liver tissues or cultured cells by lysis buffer (20 mM Tris-HCl [pH 7.5], 150 mM NaCl, 1 mM EDTA, 1 mM EGTA, 1% Triton X-100, 2.5 mM sodium pyrophosphate, 1 mM β-glycerolphosphate, 1 mM Na_3_VO_4_, 2 µg/ml leupeptin, 1 mM phenylmethylsulfonyl fluoride [PMSF]) and subjected to immunoprecipitation using antibodies as specified in the figure legends. The immunoprecipitates were then washed three times and solubilized in SDS sample buffer for further analysis.

### Western Blotting

For analysis of total cell lysates of liver, mice were euthanized after indicated treatments and liver tissues were then immediately homogenized and sonicated in a lysis buffer, centrifuged at 13,000 rpm for 15 min. For analyzing total cell lysates of cultured cells, the supernatants were collected and subjected to determination of protein concentration using Bradford assay. The lysates were then mixed with SDS sample buffer and heated at 70°C for 15 min and subjected to 8–12% SDS-PAGE and electrophoretic transfer. Immunoblots were performed according to primary antibody manufacturers’ protocols. The amount of the proteins were then analyzed using Image J analysis software and normalized against their respective controls.

### Isolation and Culture of Mouse Primary Hepatocytes

Mouse primary hepatocytes were isolated as previously described (Sun et al., 2018). Briefly, primary hepatocytes were isolated from the liver using 0.05% Collagenase Type IV (Sigma-Aldrich). Cells were then plated into six-well plates in William’s E medium supplemented with 10% FBS, 100 IU penicillin, and 100 µg/ml streptomycin. Next, primary hepatocytes were cultured in William’s E medium without serum overnight before drug treatments. For TNF-α treatment, primary hepatocytes were exposed to 50 ng/ml TNF-α for 15 min. For insulin treatment, primary hepatocytes were stimulated with 10 nM insulin for 15 min before harvesting. Cells were maintained in a 37°C incubator with 5% CO_2_.

### Enzyme-Linked Immunosorbent Assay (Elisa)

TNF-α and IL-6 were directly quantified from RAW 264.7 cells. Cells were treated with 100 ng/ml FGF-1 for 1 h prior to treatment with 100 ng/ml LPS for 12 h, and then the medium was collected from cells and subjected to TNF-α (R&D, MTA00B) or IL-6 (R&D, M6000B) ELISA kits according to the manufacturer’s instructions.

### Plasmid Constructs

Full-length complementary DNA (cDNA) encoding human TAK1 and TAB1 was obtained by PCR using human cDNA from HEK293T cells. Expression plasmids for various proteins were constructed using the pcDNA3.3 vector. All PCR products were verified by sequencing. The following primers were used for qPCR amplification: TAK1-forward, agagaattcggatccATGTCTACAGCCTCTGCCG, TAK1-Reverse,Cttccatggctcgagtcatgaagtgccttgtcgttt; TAB1-forward, agagaattcggatccATGGCGGCGCAGAGGAG, TAB1-reverse, cttccatggctcgagCTACGGTGCTGTCACCAC.

### Data Analysis

For all the data statistical analysis was performed as follows. For experiments with only two groups, the Student *t*-test was used for statistical comparisons. Analysis of variance (ANOVA) with Tukey’s post test was used to compare values among different experimental groups using GraphPad Prism 6 (La Jolla, CA). The values presented are expressed as the means ± SEM. Densitometric quantification and normalization were performed using ImageJ software. *P* < 0.05 was considered statistically significant (*) and *P* < 0.01 as highly significant (**).

## Results

### FGF-1 Improves Insulin Sensitivity in DIO Mice

**Figure 1:**
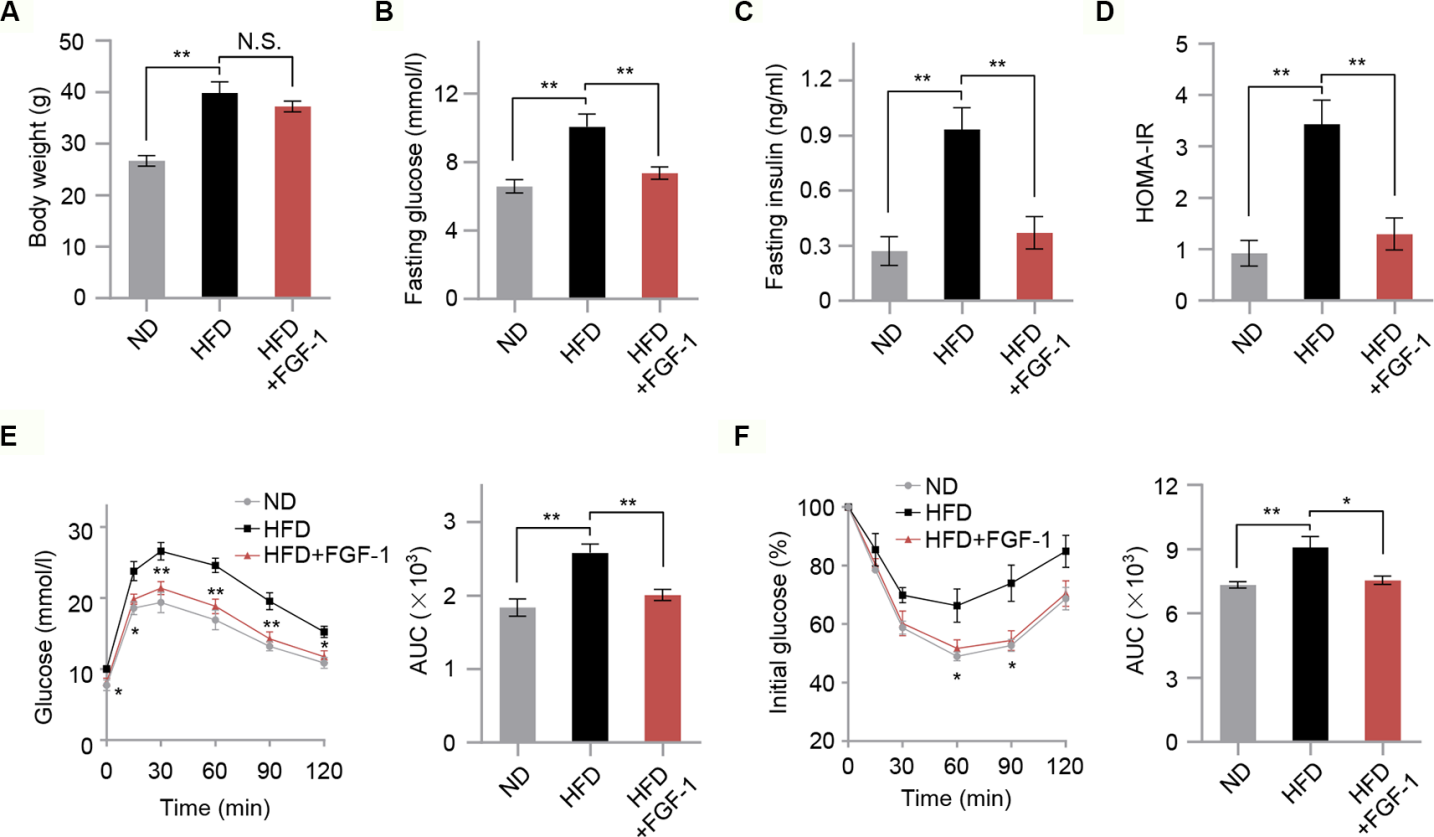
Effects of FGF-1 on insulin sensitivity in diet-induced obesity (DIO) model mice. **(A)** The body weight in mice from ND, HFD, and HFD+FGF-1 groups. Mice after subcutaneous injection of vehicle control (PBS) or FGF-1. *n* = 6 per group. **(B–D)** Overnight fasting serum glucose levels **(B)**, insulin levels **(C)**, and insulin resistance index HOMA-IR **(D)** measured from ND, HFD, and HFD+FGF-1 groups. HFD-fed mice in response to chronic treatment with 0.1 mg/kg body weight FGF-1 every day (*n* = 6 mice per group). **(E–F)** Oral glucose tolerance test (OGTT, *n* = 5 mice per group) **(E)** and insulin tolerance test (ITT, *n* = 6 mice per group) **(F)** of DIO mice after 8 weeks of FGF-1 treatment. Areas under the curve (AUC) for OGTT and ITT were calculated. DIO mice were injected subcutaneously with 0.1 mg/kg FGF-1 or PBS every day for 8 weeks. Error bars denote SEM. Statistical analysis was performed by ANOVA followed by Tukey in **(A–F)**. *P < 0.05; **P < 0.01; N.S. not significant.

To investigate the potential protective effects of FGF-1 on insulin resistance, we treated HFD-induced obesity mice with 0.1 mg/kg FGF-1 every day for 4 weeks (**Figures S1A, B**). As shown in **Figure 1**, HFD-fed mice had significantly higher levels of fasting glucose, fasting insulin, and insulin resistance index HOMA-IR compared with control ND-fed mice. Consistent with previous findings (Suh et al., 2014), FGF-1 administered by subcutaneous injection had no significant effects on body weight in DIO mice (**Figure 1A**). However, FGF-1-treated DIO mice were significantly resistant to blood glucose and insulin levels (**Figures 1B, C**). Moreover, unlike PBS-injected DIO mice, the insulin resistance index HOMA-IR of FGF-1-treated mice was markedly decreased (**Figure 1D**). Consistently, when subjected to glucose tolerance test (GTT) and insulin tolerance test (ITT), FGF-1 treated mice exhibited both greater glucose tolerance and insulin sensitivity in DIO mice (**Figures 1E, F**). Differences are indicated by the area under the curve (AUC) of GTT and ITT (**Figures 1E, F**). Importantly, FGF-1 treatment resulted in no statistically significant differences in body weight, but significantly reversed the symptoms of insulin resistance in DIO mice (**Figures 1A, E, F**).

### FGF-1 Reverses HFD-Induced Hepatic Lipid Accumulation and Insulin Resistance

**Figure 2:**
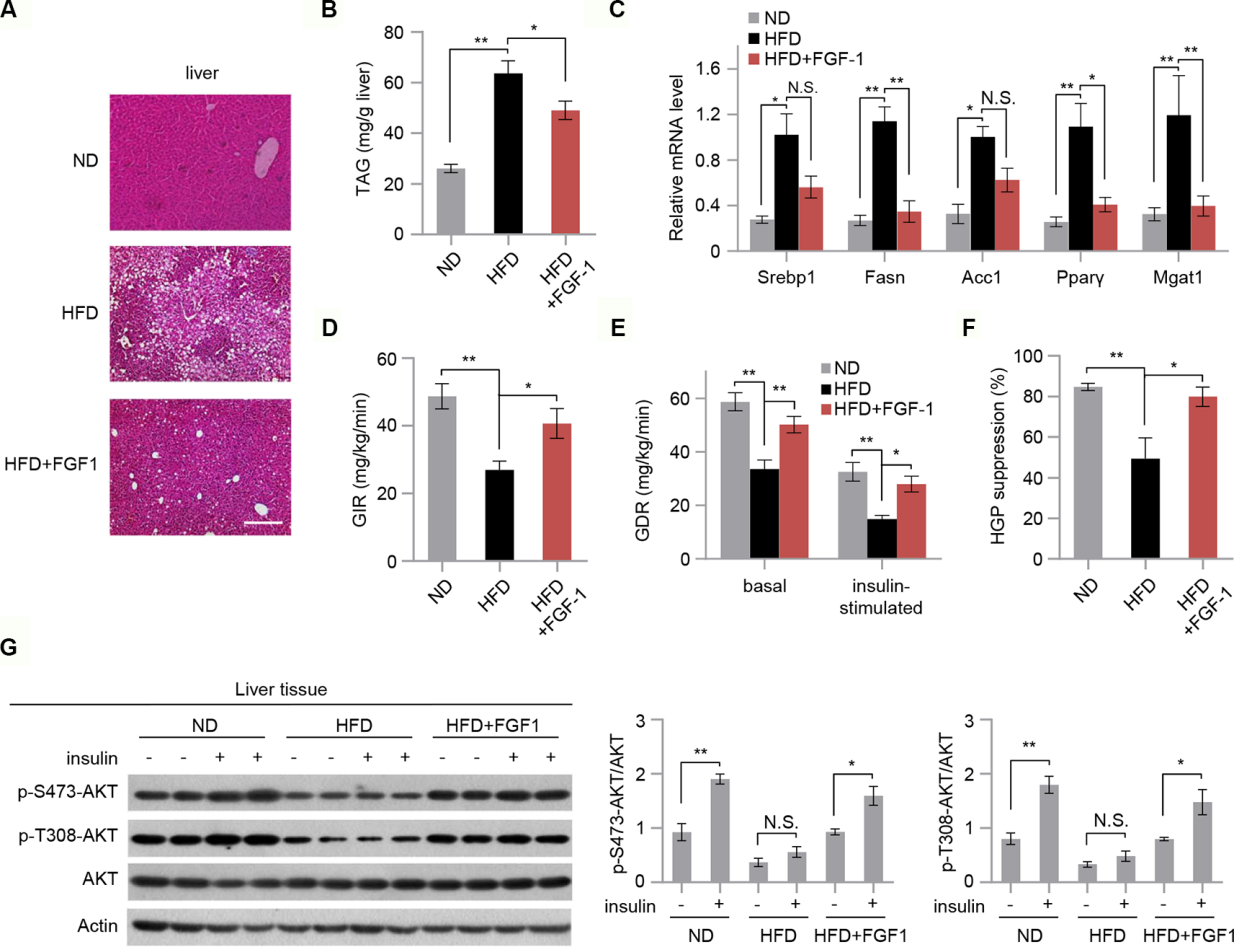
FGF-1 attenuates HFD-induced hepatic steatosis and increases hepatic insulin sensitivity. **(A)** Representative sections of liver from ND, HFD, and HFD+FGF-1 groups. DIO mice subcutaneously treated with PBS or FGF-1. Scale bar, 200 μm. **(B)** Levels of triacylglycerol (TAG) in liver tissues of ND, HFD, and HFD+FGF-1 mice (*n* = 6). **(C)** Analysis of mRNA levels of transcription factors (i.e., Srebp1, Fasn, Acc1, PPARγ, Mgat1) in livers from ND, HFD, and HFD+FGF-1 group (*n* = 6). **(D–F)** Hyperinsulinemic-euglycemic clamp study in DIO mice after 8 weeks of PBS (*n* = 6) or FGF-1 (*n* = 6) administration. Glucose infusion rate (GIR, **D**), glucose disposal rate (GDR, **E**), and percent suppression of HGP **(F)** are shown. **(G)** Western blot analysis and quantification of AKT phosphorylation in liver after intraperitoneal injection of insulin or saline in ND, HFD, and HFD+FGF-1 groups. All mice were fasted 6 h before insulin or saline injection. Error bars denote SEM. Statistical analysis was performed by ANOVA followed by Tukey in **(B–G)**. *P < 0.05; **P < 0.01; N.S. not significant. Uncropped blots can be found in **Supplementary Figure 5**.

Hepatic steatosis is a strong predictor of the development of insulin resistance (Perry et al., 2014). We then assessed the effect of FGF-1 on HFD-induced hepatic steatosis. Although there were no significant changes in body weight, chronic treatment with FGF-1 slightly decreased liver weights albeit no statistical significant difference in DIO mice (**Figure 1A** and **Figures S2A, B**). Histological analysis of liver sections has revealed cytoplasmic change, the extensive existence of micro- and macrovesicular hepatocyte vacuolation, and severe hepatic steatosis in HFD-fed mice, as evidenced by H&E staining and quantitative analyses (**Figures 2A, B**). Unexpectedly, administration of FGF-1 effectively reduced hepatocellular vacuolation and significantly improved hepatic steatosis in HFD-fed mice (**Figures 2A, B**). Consistently, FGF-1 treatment resulted in a significant reduction in the mRNA levels of lipogenic genes (Srebp1, Fasn, Acc1, Pparγ, and Mgat1) in DIO mice (**Figure 2C**).

To further investigate the vital role of FGF-1 in insulin sensitivity, we performed hyperinsulinemic-euglycemic clamp studies in DIO mice. The steady-state glucose infusion rate (GIR) was increased in chronic FGF-1-treated DIO mice, reflecting an increased responsiveness to insulin (**Figure 2D**). Meanwhile, treatment with FGF-1 increased whole-body and insulin-stimulated glucose disposal rates (GDR) (**Figure 2E**). Furthermore, FGF-1-treated mice showed marked improvements in the ability of insulin to suppress hepatic glucose production (HGP), revealing increased hepatic insulin sensitivity as well (**Figure 2F** and **Figure S2C**). In addition, insulin stimulation increased phosphorylation of AKT at T308 and S473 in liver isolated from ND, HFD, and HFD+FGF-1 mice, and HFD feeding inhibited insulin-stimulated phosphorylation of AKT. Importantly, the inhibitory effect of the HFD on AKT phosphorylation was absent in FGF-1 treated HFD mice (**Figure 2G**).

### FGF-1 Exerts Anti-Inflammatory Effects in DIO Mice

**Figure 3:**
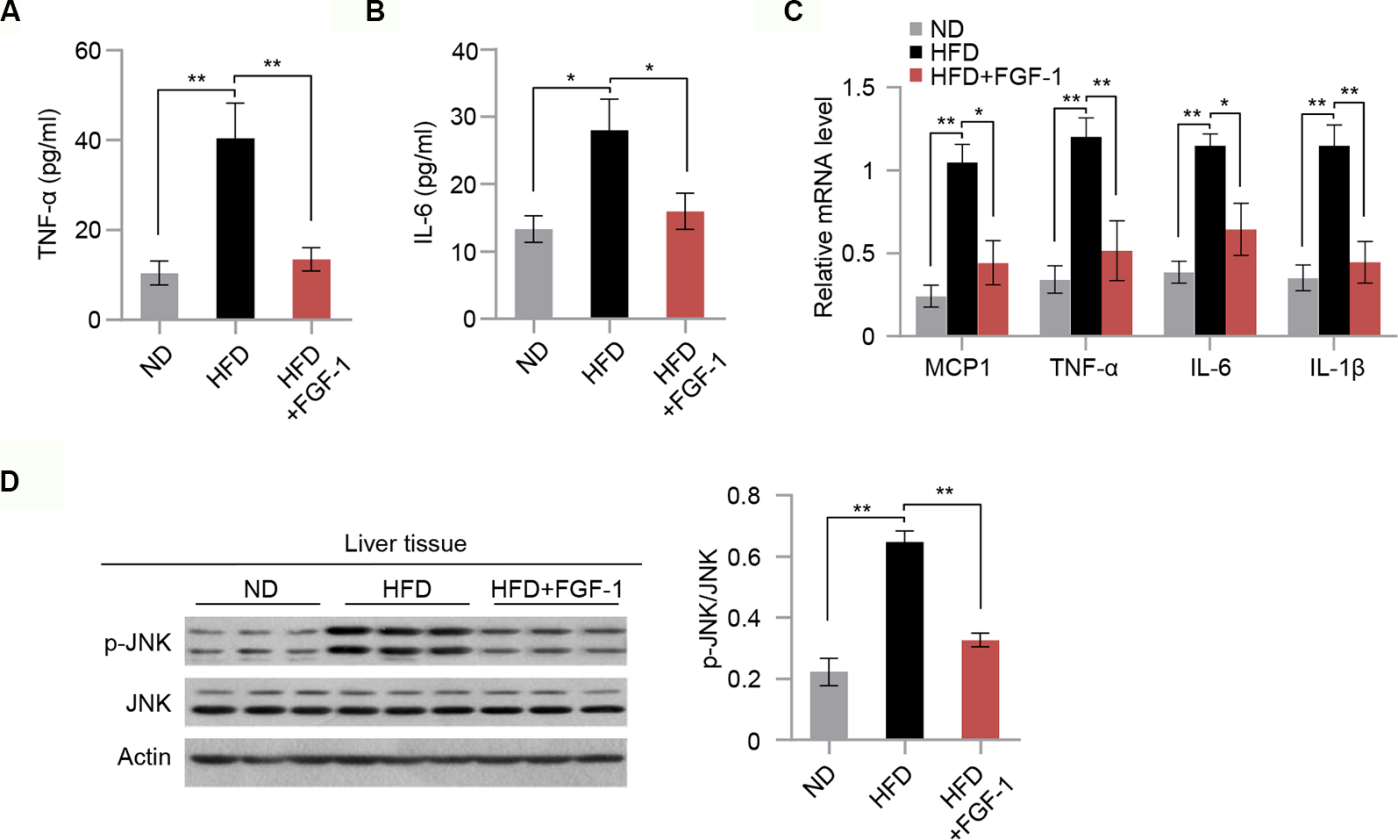
Potential anti-inflammatory action of FGF-1 in DIO mice. **(A–B)** Plasma concentrations of pro-inflammatory cytokines TNF-α **(A)** and IL-6 **(B)** in ND, HFD, and HFD+FGF-1 group. **(C)** Relative mRNA levels of hepatic inflammation-related gene expression in liver tissues of ND, HFD, and HFD+FGF-1. **(D)** Western blot analysis of total liver lysates from ND, HFD, and HFD+FGF-1 groups. Error bars denote SEM. Statistical analysis was performed by ANOVA followed by Tukey in **(A–D)**. *P < 0.05; **P < 0.01. Uncropped blots can be found in **Supplementary Figure 5**.

It was previously reported that chronic inflammation is closely associated with insulin resistance (Shoelson et al., 2006). Next, we explored the mechanisms of the protective action of FGF-1 against insulin resistance. Consistent with previous reports (Suh et al., 2014; Liang et al., 2018), we found that FGF-1 treatment markedly repressed the ability of HFD to stimulate cytokine secretion (TNF-α and IL6) in the serum (**Figures 3A, B**). Moreover, mRNA expression of inflammatory genes were significantly inhibited in the liver tissue of FGF-1-injected DIO mice (**Figure 3C**). The c-Jun N-terminal kinases are critical determinants of obesity-associated inflammation and insulin resistance (Hirosumi et al., 2002; Han et al., 2013). Thus, we examined whether FGF-1 regulates JNK activity in DIO mice. In liver tissues of DIO mice, FGF-1 markedly inhibited HFD-induced phosphorylation of JNK, while downstream effects on AKT phosphorylation were significantly enhanced (**Figures 2G** and **3D**).

### FGF-1 Ameliorates TNF-A-Induced Insulin Resistance *In Vitro* and *In Vivo*

**Figure 4:**
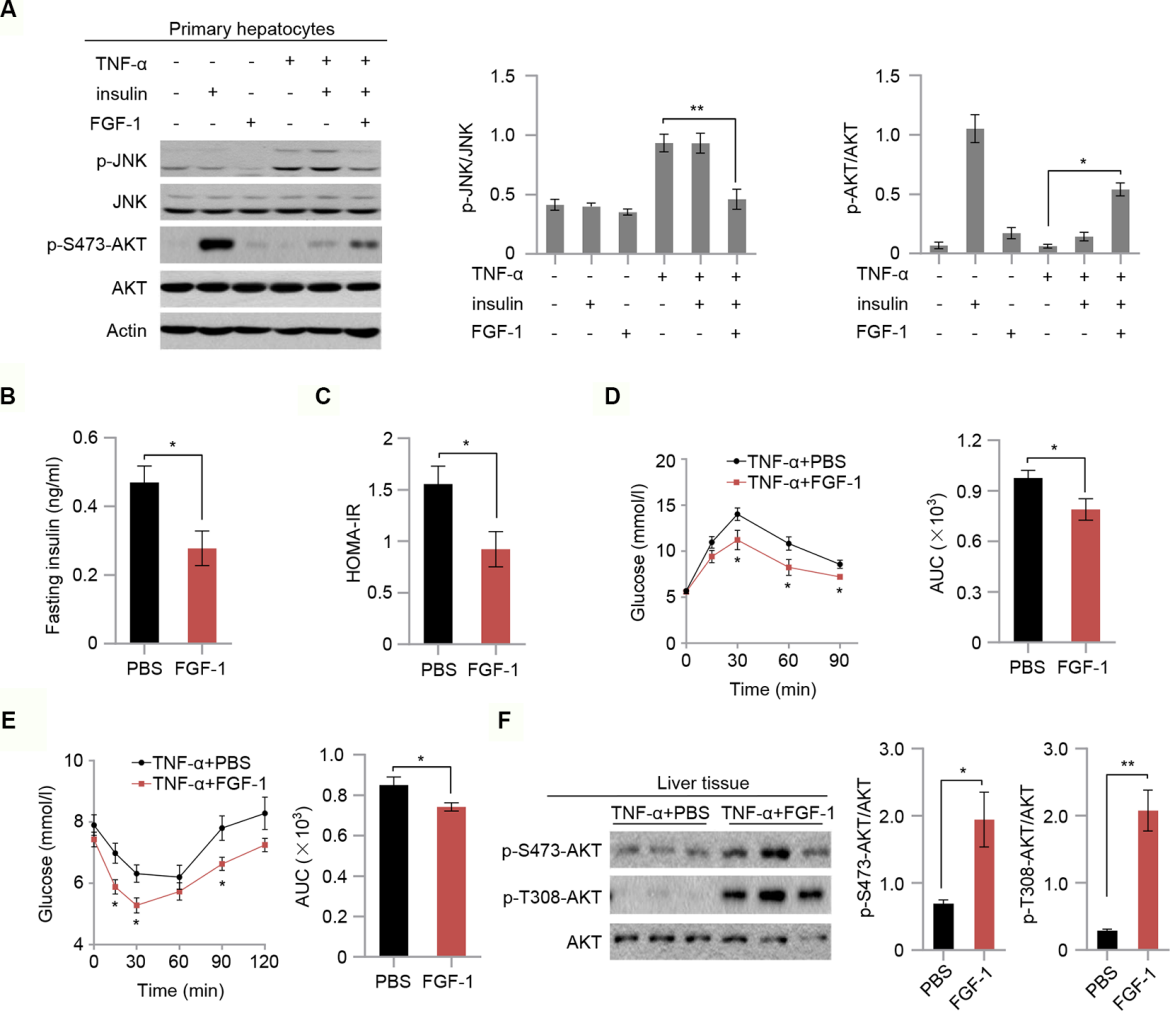
FGF-1 restores TNF-α-induced insulin resistance *in vitro* and *in vivo.* **(A)** Western blot analysis of total cell lysates from primary hepatocytes isolated from wild type C57BL/6J mice. Primary hepatocytes were treated with 100 ng/ml FGF-1 or control for 1 h prior to 50 ng/ml TNF-α or 10 nM insulin for 15 min. Experiments in this figure were performed three times. **(B–C)** Fasting serum insulin levels **(B)** and insulin resistance index HOMA-IR **(C)** of TNF-α-induced insulin resistance mice after treatment with 0.5 mg/kg body weight FGF-1 every day (*n* = 6 mice per group). **(D–E)** OGTT (*n* = 6 mice per group) **(D)** and ITT (*n* = 6 mice per group) **(E)** of TNF-α-induced insulin resistance mice. AUC was calculated for OGTT and ITT. TNF-α-induced insulin resistant mice were treated with FGF-1 (0.5 mg/kg) every day. **(F)** Western blot analysis of total liver lysates from TNF-α-induced insulin resistance mice treated with FGF-1 or control. Error bars denote SEM. Statistical analysis was performed with a two-tailed unpaired Student’s *t*-test in **(B–F)**. *P < 0.05; **P < 0.01. Uncropped blots can be found in **Supplementary Figure 5**.

To test whether FGF-1 attenuates TNF-α-triggered insulin resistance, we next investigated the role of FGF-1 in TNF-α-induced hepatic insulin resistance. As expected, FGF-1 restores reduced phosphorylation of JNK and reversed changes in the insulin signaling pathway as evidenced by elevated AKT phosphorylation, which is induced by TNF-α treatment in mouse primary hepatocytes (**Figure 4A**). To further assess the role of FGF-1 in TNF-α-induced hepatic insulin resistance *in vivo*, we injected FGF-1 into wild-type mice treated with TNF-α. Treatment with FGF-1 had no significant effects on body weight, fasting glucose (**Figures S3A, B**). Meanwhile, no difference in the liver histopathology and hepatic lipid accumulation in TNF-α-induced hepatic insulin resistance mice with FGF-1 treatment (**Figures S3C, D**); however, FGF-1 ameliorated TNF-α-induced insulin resistance, as assessed by decreased insulin levels and reduced insulin resistance index HOMA-IR (**Figures 4B, C**). Similarly, GTT and ITT results indicated restored glucose tolerance and improved insulin sensitivity upon FGF-1 injection into mice treated with TNF-α (**Figures 4D, E**). Moreover, FGF-1 reversed changes in the insulin signaling pathway induced by TNF-α (**Figure 4F**).

### FGF-1 Mediates Anti-Inflammatory Effects

**Figure 5:**
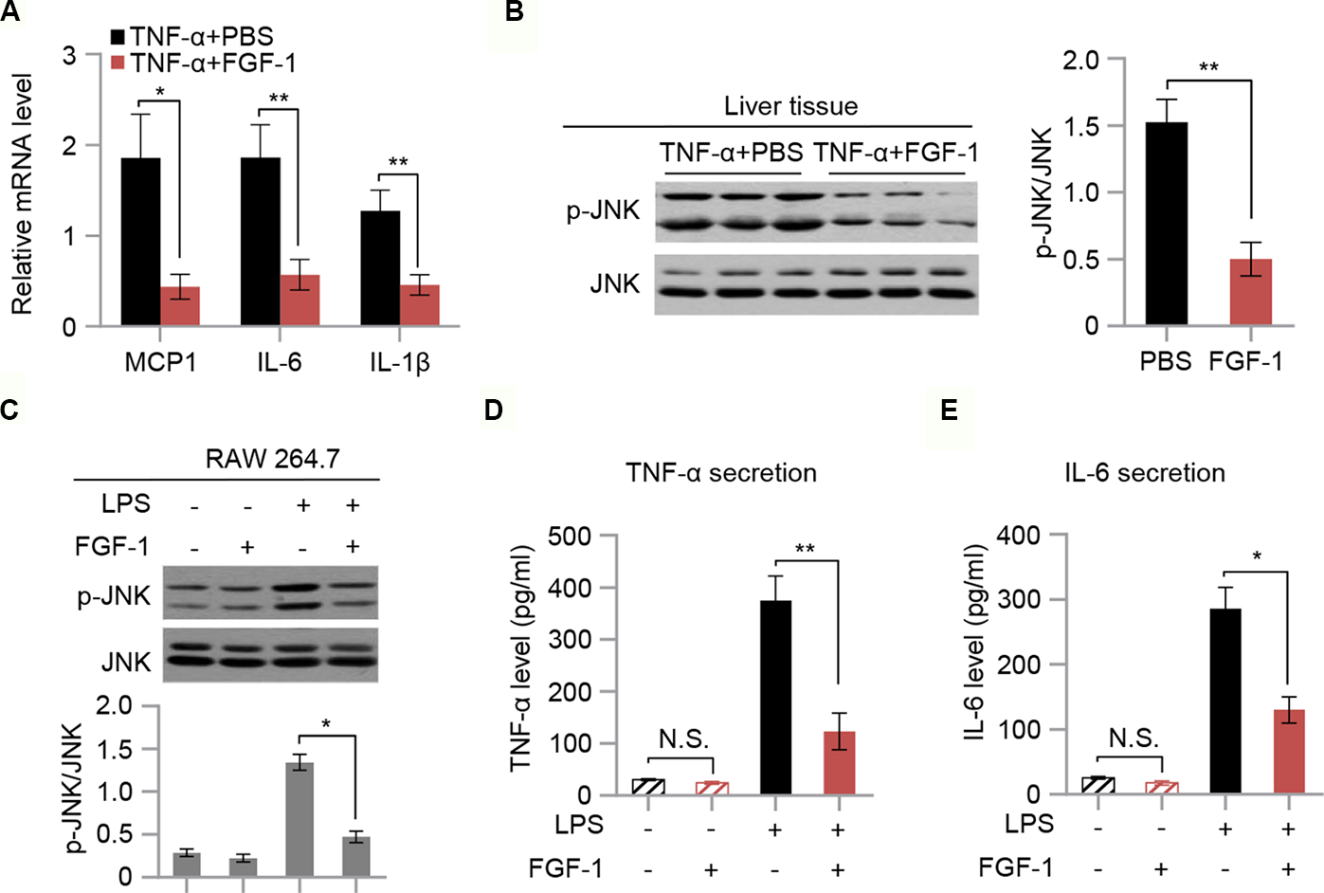
FGF-1 reduces TNF-α-induced inflammation and LPS-induced cytokine secretion. **(A)** Relative mRNA levels of hepatic inflammatory-related gene expression in liver tissue of TNF-α-induced insulin resistant mice in response to treatment with FGF-1. **(B)** Western blot analysis of liver lysates from TNF-α-induced insulin resistant mice treated with FGF-1 or control. **(C)** RAW 264.7 cells were treated with 100 ng/ml FGF-1 for 1 h prior to 100 ng/ml of LPS treatment for 10 min, and then their lysates were subjected to western blot. Experiments in this figure were performed three times. **(D–E)** Cytokine secretion was measured in RAW 264.7 cells by ELISA. Error bars denote SEM. Statistical analysis was performed by two-tailed unpaired Student’s *t*-test in **(B)** or by ANOVA followed by Tukey in **(A)** and **(C–E)**. *P < 0.05; **P < 0.01, N.S. not significant. Uncropped blots can be found in **Supplementary Figure 5**.

To evaluate the effect of FGF-1 on TNF-α-triggered inflammation, mRNA levels of MCP1, IL-6, and IL-1β in liver tissues of TNF-α-treated mice were measured. Our findings indicated that mRNA expression of inflammatory mediators (MCP1, IL-6, and IL-1β) were markedly inhibited in response to FGF-1 (**Figure 5A**). Moreover, western blot analysis indicated that FGF-1 effectively prevented increased JNK phosphorylation in the liver tissue of TNF-α-treated mice (**Figure 5B**). Next, we investigated whether FGF-1 directly modulates inflammation in macrophages. To examine this, we pretreated RAW 264.7 cells with FGF-1 for 1 h, followed by LPS stimulation. Importantly, FGF-1 strongly inhibited LPS-induced phosphorylation of JNK, cytokine secretion, and levels of inflammatory gene expression in RAW 264.7 cells (**Figures 5C–E** and **Figures S4A–C**).

### FGF-1 Attenuates Inflammation and Insulin Resistance Through Blocking the TAK1/TAB1 Interaction

**Figure 6:**
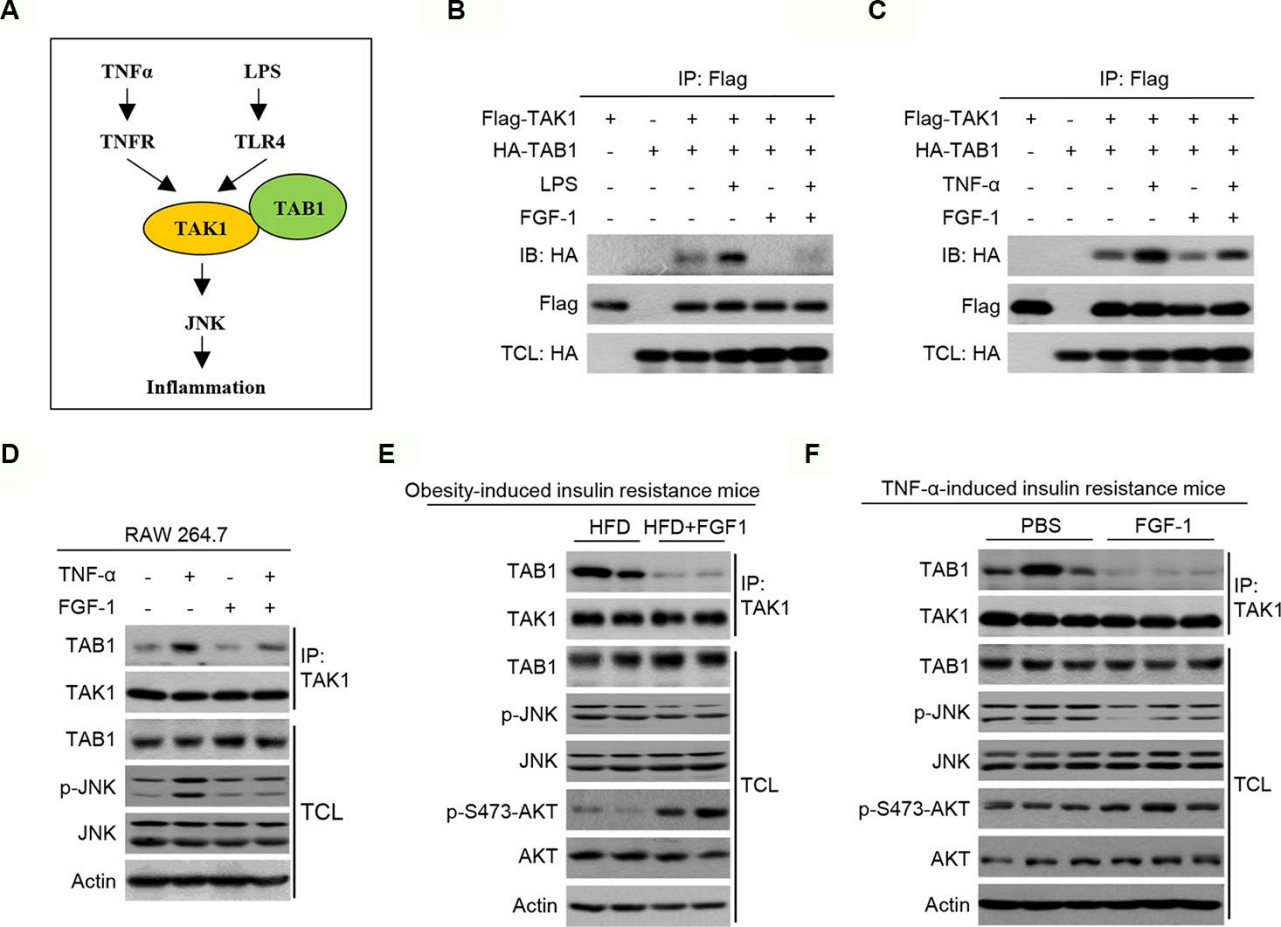
Potential anti-inflammatory mechanism of FGF-1 in insulin resistance. **(A)** Schematic diagram of TAK1-TAB1-mediated JNK signaling pathway being essential for regulating inflammation. **(B–C)** The interaction between TAK1 and TAB1 was detected by co-immunoprecipitation in HEK293T cells. **(D)** Total cell lysates from RAW 264.7 cells treated with 10 ng/ml FGF-1 or control for 1 h prior to 50 ng/ml TNFα for 15 min were immunoprecipitated with an antibody to TAK1, followed by immunoblotting. **(E–F)** Total liver lysates from mice treated with FGF-1 or control were immunoprecipitated with an antibody to TAK1, followed by immunoblotting. TCL, total cell lysate. Uncropped blots can be found in **Supplementary Figure 5**.

TNF-α/LPS activates inflammation through the transforming growth factor-β activated kinase 1 (TAK1)-JNK pathway (**Figure 6A**). Furthermore, LPS or TNF-α activates TAK1 by promoting TAK1’s association with TAK1 binding protein 1 (TAB1). As previously reported, FGF-1 significantly inhibits TNF-α-mediated inflammatory responses (Liang et al., 2018). Since the TNF-α-TNFR and LPS-TLR4 signaling cascades converge downstream of FGF-1, these results indicate that the site of FGF-1-induced inhibition is upstream of JNK. Consequently, we investigated whether FGF-1 inhibited the TAK1/TAB1 association. In HEK293T cells treated with TNF-α or LPS, the interaction between TAK1 and TAB1 was strongly increased (**Figures 6B, C**). Importantly, FGF-1 treatment largely abrogated the TAK1/TAB1 association (**Figures 6B, C**). Similar results were obtained in RAW264.7 cells, where there was an observed decrease in interaction between endogenous TAK1 and TAB1 in response to FGF-1 treatment (**Figure 6D**). In addition, interaction between endogenous TAK1 and TAB1 was strongly inhibited in the liver of FGF-1 treated obesity- or TNF-α-induced insulin resistance mice (**Figures 6E, F**).

## Discussion

Insulin resistance may be part of metabolic syndrome, and it has been associated with a higher risk of developing diabetes (Kahn et al., 2006; Laakso and Kuusisto, 2014; Czech, 2017). There are many causes of insulin resistance, including metabolic syndrome, stress, abdominal obesity, and inflammation (Kahn et al., 2006; Shoelson et al., 2006; Asrih and Jornayvaz, 2015). Several observations provide evidence that FGF-1 treatment significantly improved insulin resistance (Suh et al., 2014; Huang et al., 2017). However, the protective effect of FGF-1 in insulin resistance have not been fully understood. In this study, we demonstrated that FGF-1 treatment significantly ameliorated obesity and TNF-α-induced stimulated cytokine secretion such as TNF-α and IL-6 as well as enhancing anti-inflammatory. Mechanistically, FGF-1 ameliorated insulin resistance and inflammation was accompanied by decreased JNK signaling. In addition, FGF-1 regulates JNK phosphorylation *via* the TAK1-TAB1 association.

In this study, we observed that administration of FGF-1 improved insulin resistance as well as metabolic disorder. In addition, FGF-1 treatment significantly inhibits hepatic lipid accumulation and reduced the HFD-induced increases in plasma glucose and insulin, and exhibited greater glucose tolerance and insulin sensitivity in DIO mice. This is in agreement with a previous study that showed that FGF-1 regulates glucose metabolism and improvement of hepatosteatosis (Suh et al., 2014; Huang et al., 2017). We also found that the body weight of FGF-1-treated HFD mice did not decrease in comparison with PBS-treated HFD-fed mice. As previously reported (Sasaki et al., 1994; Li et al., 1998; Suzuki et al., 2001), the temporary body weight-lowering effect of FGF-1 is dependent on a transient suppression of food intake. However, injection of FGF-1 similarly reduced HFD-induced glucose and insulin levels and improved insulin resistance under fasting conditions, which eliminated the effect of individual body weight on insulin sensitivity.

Obesity-associated with chronic systemic inflammation is responsible for the development of insulin resistance, which makes obesity a major risk factor for insulin resistance (Hirosumi et al., 2002; Kahn et al., 2006; Chen and Chen, 2015). Obesity might increase the expression of some inflammatory cytokines and activate several inflammation-related signaling pathways, both of which are involved in the pathogenesis of insulin resistance by inhibiting insulin signaling and action (Dandona et al., 2004; Shoelson et al., 2006). In addition, obesity causes lipid accumulation, activates JNK and NF-κB signaling pathways, and might subsequently increase the production of pro-inflammatory cytokines such as TNF-α and IL-6 (Shoelson et al., 2006). It has been shown that FGF-1 plays a key role in nutrient homeostasis (Jonker et al., 2012; Suh et al., 2014), and *Fgf-1^-/-^* mice develop severe insulin resistance after HFD feeding (Jonker et al., 2012). In particular, pharmacological treatment with recombinant FGF-1 increases insulin-dependent glucose uptake and suppresses hepatic production of glucose to achieve whole-body insulin sensitization (Suh et al., 2014; Huang et al., 2017). Liang *et al.* (Liang et al., 2018) recently reported that intraperitoneal administration of FGF-1 into both type 1 and type 2 diabetes models significantly suppressed inflammation. Importantly, FGF-1-mediated inhibition of renal inflammation *in vivo* was accompanied by attenuated JNK and NF-κB signaling pathways (Liang et al., 2018). In this present study, obesity triggered a robust systemic inflammatory profile were significantly inhibited by FGF-1. Thus, FGF-1 ameliorated insulin resistance both in obesity induced insulin resistance mice, which is associated with its anti-inflammatory action and not through its glucose-lowering activity.

Consistent with previous reports, FGF-1 was able to correct elevated blood glucose levels in DIO mice (Suh et al., 2014). High glucose levels activate the JNK signaling pathway, which is an important player in inflammation and regulates the transcription of a number of inflammatory cytokines (Hirosumi et al., 2002; Sabio and Davis, 2010; Liang et al., 2018). Therefore, we next examined the effects of FGF-1 on mice treated with TNF-α to induce insulin resistance (Hotamisligil et al., 1994; Peraldi et al., 1996). We observed that FGF-1 treatment significantly improved insulin resistance in TNF-α-induced insulin resistance *in vitro* and *in vivo*. These results indicated that FGF-1 likely ameliorates insulin resistance *via* a mechanism that is independent of its glucose-lowering activity. Thus, we speculate that the anti-inflammatory effect of FGF-1 is strong and able to block inflammation in TNF-α-induced insulin resistance, resulting in similar protective effects in both models.

In summary, the present study confirms the protective effects of FGF-1 in DIO- or TNF-α-induced insulin resistance mice. Mechanistically, FGF-1 attenuates inflammation and insulin resistance through blocking the TAK1/TAB1 interaction. Our findings also expand the knowledge of physiological functions of FGF-1 in insulin resistance and suggest inhibition of inflammation as an attractive therapeutic strategy for the treatment of insulin resistance.

## Data Availability Statement

All datasets generated for this study are included in the article/**Supplementary Material**.

## Ethics Statement

The animal study was reviewed and approved by The Institutional Animal Care and Use Committee of Wenzhou Medical University.

## Author Contributions

YH and LS conceived the project and designed the experiments. LF, LD, JL, JN, and LS performed the experiments and participated in discussion of the results. JN, YH, and LS helped conceive the project and interpret the data. LF, YH, and LS analyzed the data and wrote the manuscript.

## Funding

This work was supported by the Natural Science Foundation of Zhejiang Province LQ17H070001 (to LF) and the Science and Technology Project of Jinhua 2018-4-036 (to LF) and 2019-3-005 (to LD).

## Conflict of Interest

The authors declare that the research was conducted in the absence of any commercial or financial relationships that could be construed as a potential conflict of interest.
