# Supplementary material for: Fibroblast Growth Factor-1 Improves Insulin Resistance via Repression of JNK-Mediated Inflammation
Source: Front Pharmacol. 2019 Dec 5;10:1478. doi: 10.3389/fphar.2019.01478 (PMC6906192; doi:10.3389/fphar.2019.01478)

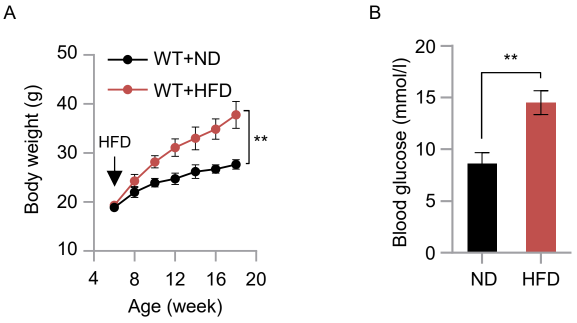

Figure S1 Related to Figure 1. Validation of HFD-induced obesity mice.
A. Body weight of wild-type C57BL/6J male mice fed with a normal chow diet (ND) (n = 7 per group) or high-fat diet (HFD, starting at 6 weeks of age) (n =30 per group). B. Levels of blood glucose in WT ND (n = 7 per group) or HFD (n =30 per group) male mice. Error bars denote SEM. Statistical analysis was performed by two-tailed unpaired Student's *t*-test in **A** and **B**. $^{**}P < 0.01$.

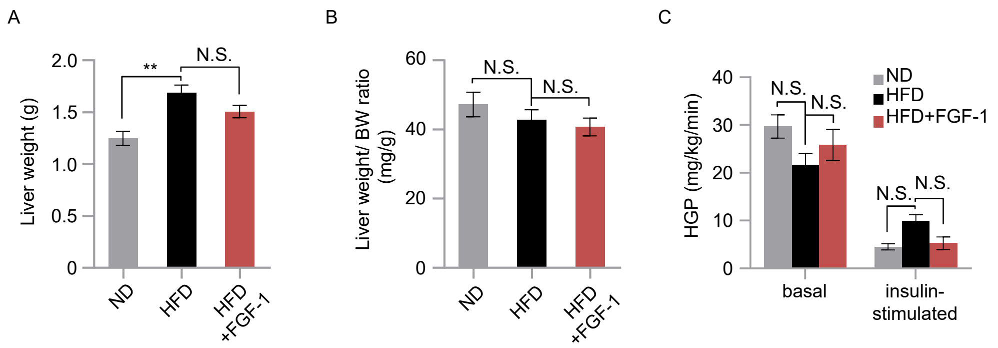

Figure S2 Related to Figure 2. FGF-1 improves hepatic insulin sensitivity

A. The liver weight in mice from ND, HFD and HFD+FGF-1 groups. Mice after subcutaneous injection of vehicle control (PBS) or FGF-1. *n* = 6 per group. B. The liver-BW ratio in ND, HFD and HFD+FGF-1 mice. C. Basal and insulin-stimulated hepatic glucose production (HGP) rate measured during hyperinsulinemic-euglycemic clamp study on ND, HFD and HFD+FGF-1 groups. *n* = 6 per group. Error bars denote SEM. Statistical analysis was performed by ANOVA followed by Tukey in **A-C**. $^{**}P < 0.01$; N.S. not significant.

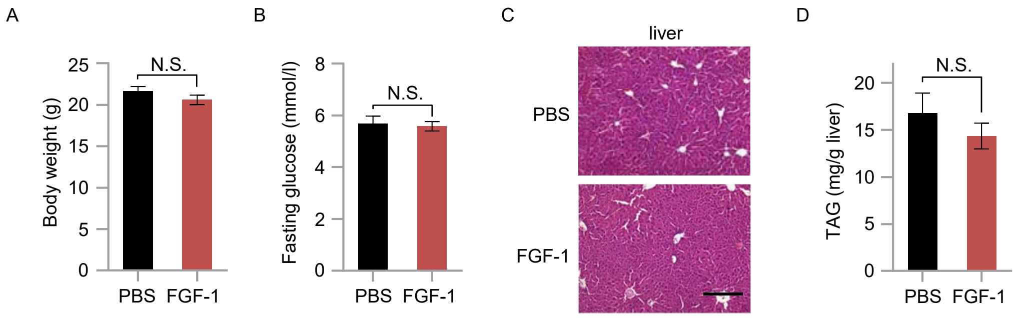

Figure S3 Related to Figure 4. FGF-1 restores TNF-α-induced insulin resistance *in vivo.*
A. Body weight of TNF-α-induced insulin resistance mice after subcutaneous injection of control PBS or FGF-1. n = 6 per group. B. Overnight fasting serum glucose level of TNF-α-induced insulin resistance mice after treatment with 0.5 mg/kg body weight FGF-1 every day (n = 6 mice per group). C. Representative sections of liver from TNF-α-induced insulin resistance mice subcutaneously treated with PBS or FGF-1. Scale bar, 200 µm. D. Liver TAG level of TNF-α-induced insulin resistance mice (n = 6 mice per group). Error bars denote SEM. Statistical analysis was performed by two-tailed unpaired Student's t-test in **A**, **B** and **D**. N.S. not significant.

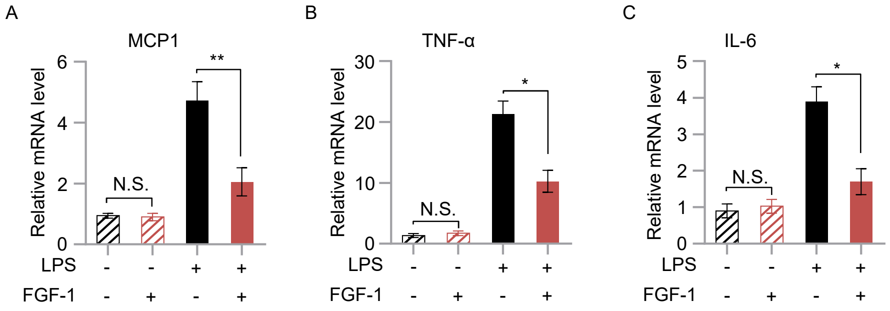

Figure S4 Related to Figure 5. FGF-1 reduced LPS-induced inflammatory gene mRNA expression level.

(A-C) FGF-1 inhibits LPS-induced inflammatory gene (MCP1,TNF-α and IL-6,) mRNA expression level in RAW 264.7 cells. Error bars denote SEM. Statistical analysis was performed by ANOVA followed by Tukey. *$P < 0.05$; * *$P < 0.01$; N.S. not significant.

Supplementary Figure 5. Uncropped blots with size marker indications

Figure 2G

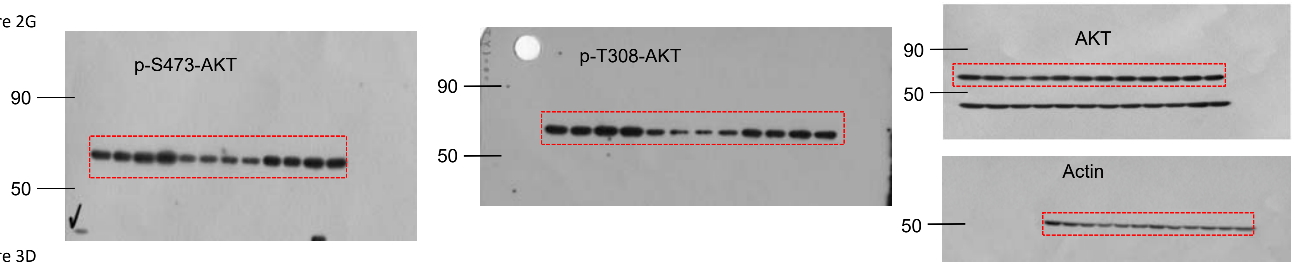

Figure 3D

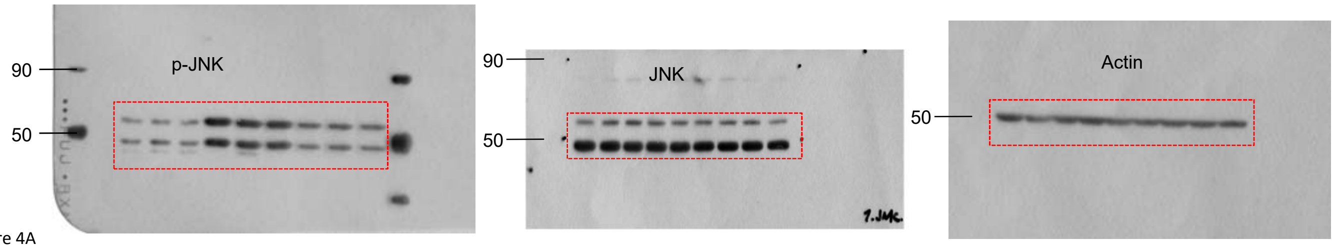

Figure 4A

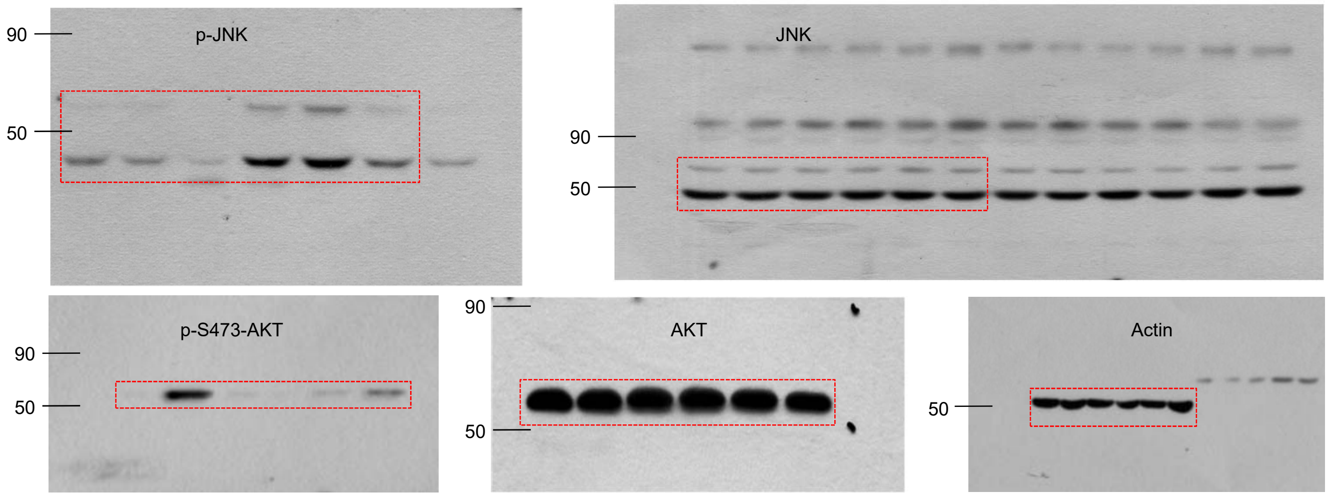

Figure 4F

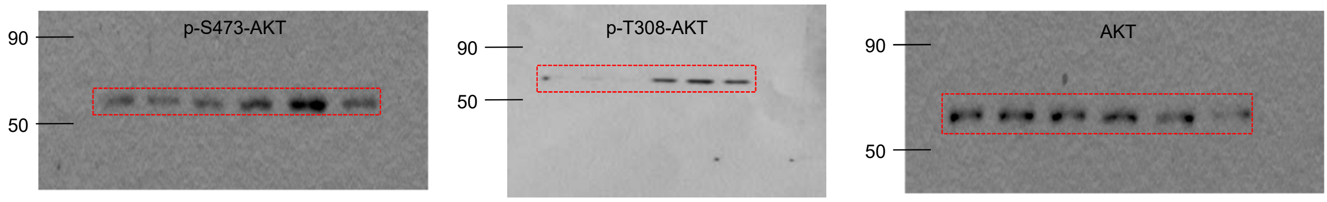

Figure 5B

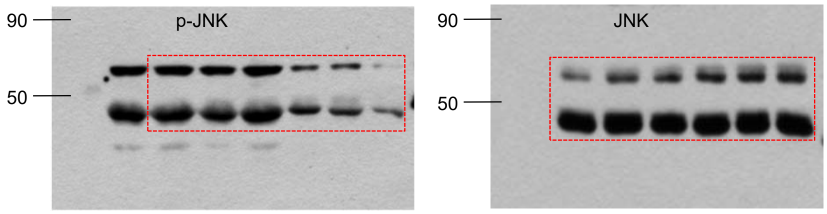

Figure 5C

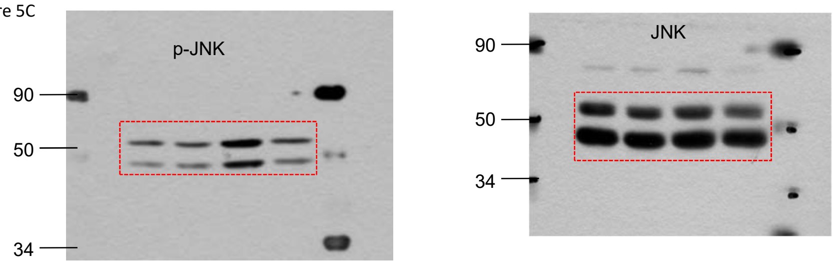

Supplementary Figure 5. Uncropped blots with size marker indications (continued)

Figure 6B

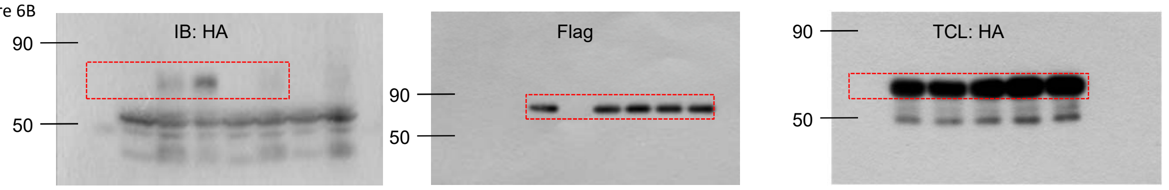

Figure 6C

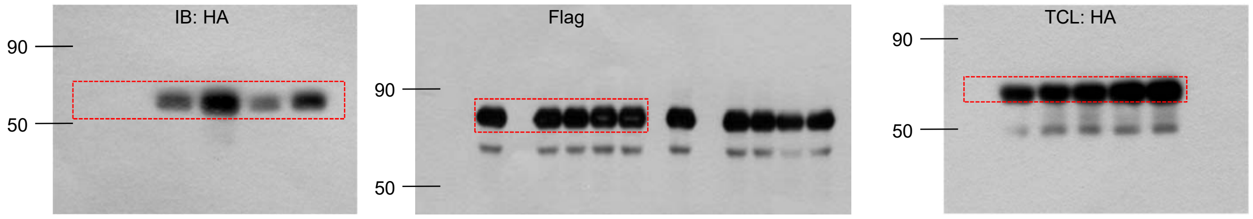

Figure 6D

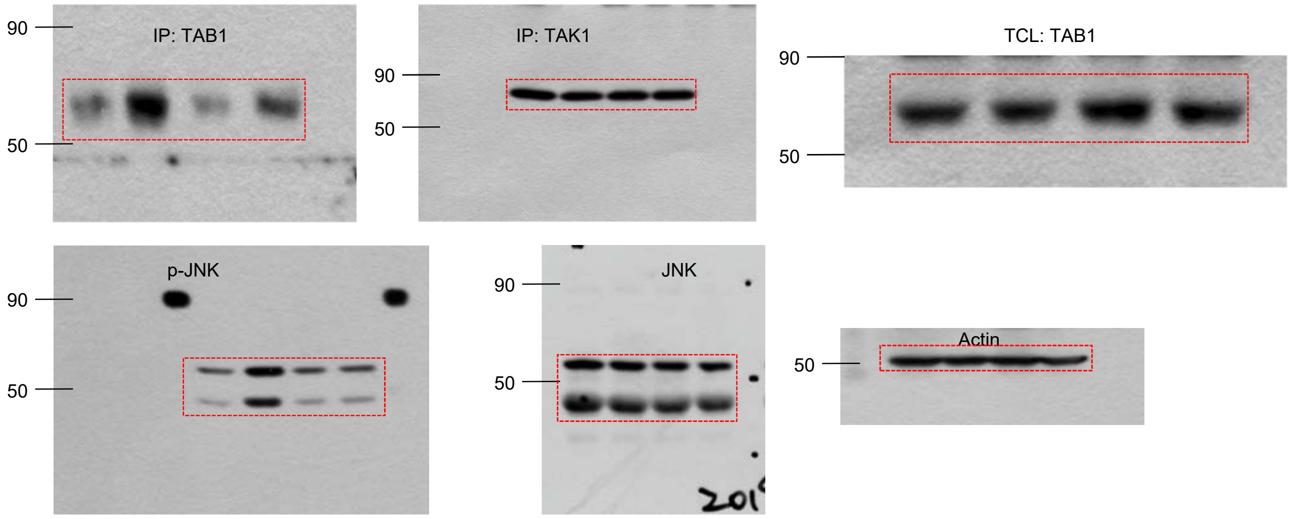

Figure 6E

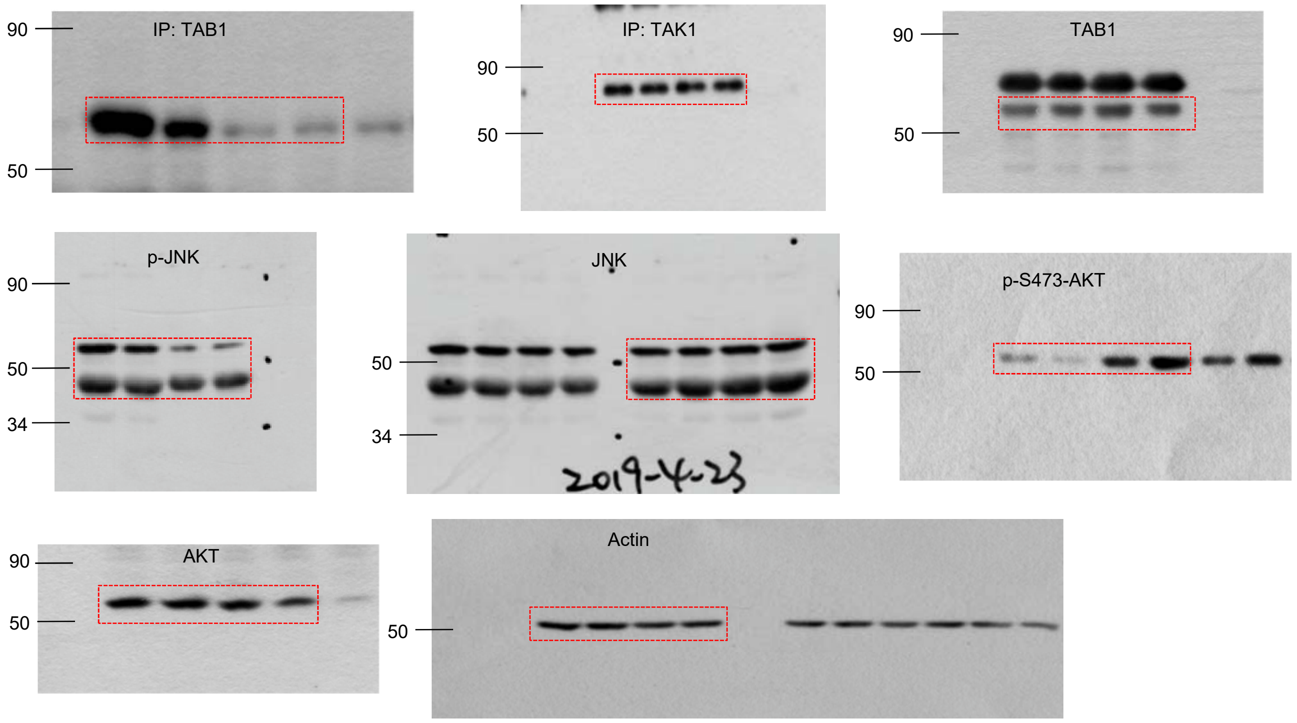

Supplementary Figure 5. Uncropped blots with size marker indications (continued)

Figure 6F

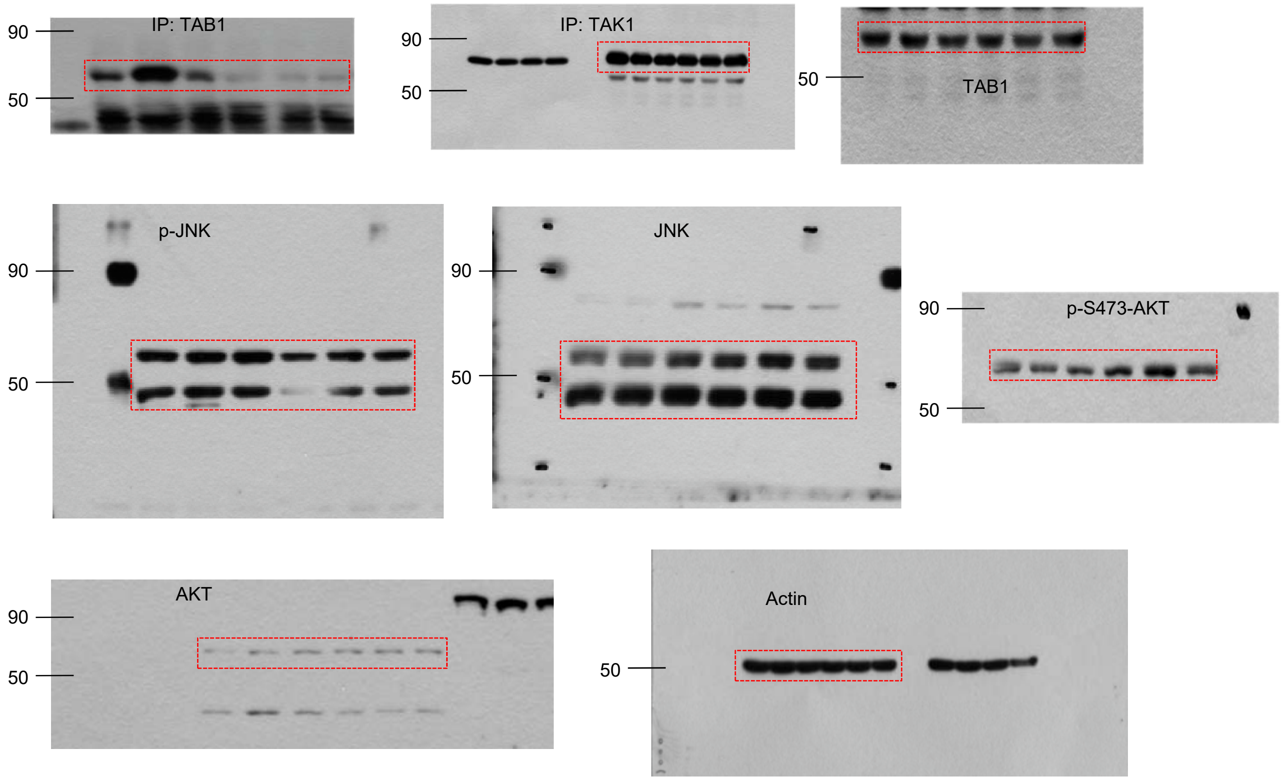

Supplement: Supplementary file 1 [file DataSheet_1.pdf]
