# Supplementary material for: Fibroblast Growth Factor-1 Improves Insulin Resistance via Repression of JNK-Mediated Inflammation
Source: Front Pharmacol. 2019 Dec 5;10:1478. doi: 10.3389/fphar.2019.01478 (PMC6906192; doi:10.3389/fphar.2019.01478)
Supplement: Supplementary file 2 [file Table_1.docx]

**Table S1. Primer sequences for real-time PCR**

| **Species** | **Gene** | **Forward (5’ to 3’)** | **Reverse (5’ to 3’)** |
| --- | --- | --- | --- |
| mouse | Srebp1 | GCAGCCACCATCTAGCCTG | CAGCAGTGAGTCTGCCTTGAT |
| mouse | Fasn | GGAGGTGGTGATAGCCGGTAT | TGGGTAATCCATAGAGCCCAG |
| mouse | Acc1 | ATGGGCGGAATGGTCTCTTTC | TGGGGACCTTGTCTTCATCAT |
| mouse | Pparγ | TCGCTGATGCACTGCCTATG | GAGAGGTCCACAGAGCTGATT |
| mouse | Mgat1 | TTGTGCTTTGGGGTGCTATCA | CCACAGTGGGAACTCTCCA |
| mouse | MCP1 | TTAAAAACCTGGATCGGAACCAA | GCATTAGCTTCAGATTTACGGGT |
| mouse | TNF-α | GACGTGGAACTGGCAGAAGAG | TTGGTGGTTTGTGAGTGTGAG |
| mouse | IL-6 | CCAAGAGGTGAGTGCTTCCC | CTGTTGTTCAGACTCTCTCCCT |
| mouse | IL-1β | GCAACTGTTCCTGAACTCAACT | ATCTTTTGGGGTCCGTCAACT |
| mouse | Actin | TTGTAACCAACTGGGACGATATGG | CGACCAGAGGCATACAGGGACAAC |
